# Translating Senolytics From Mice to Humans

**DOI:** 10.1093/geroni/igaf122.679

**Published:** 2025-12-31

**Authors:** Sundeep Khosla

**Affiliations:** Mayo Clinic College of Medicine and Science, Rochester, Minnesota, United States

## Abstract

Extensive studies in rodents have shown that senescent cells accumulate across tissues and their clearance leads to improvements in healthspan and lifespan. Based on these studies, there is intense interest in translating these findings into humans. Based on ClinicalTrials.gov, there are currently 26 ongoing studies on “senolytics” and 32 on “fisetin”. However, to date, there are only 9 published clinical trials of senolytics, only 2 of which included a control group. Collectively, the initial findings from these trials have indicated possible biological efficacy of senolytics (dasatinib + quercitin) in improving biomarkers of healthspan in humans with no significant safety concerns. However, these initial studies also highlight the potential heterogeneity of aging mechanisms in humans and argue for a more personalized approach in future senolytic trials. Specifically, it is likely going to be critical to identify aging individuals with a sufficiently high burden of senescent cells that would result in a beneficial effect from the senolytic intervention.

